# Long Non-Coding RNAs: New Players in Hematopoiesis and Leukemia

**DOI:** 10.3389/fmed.2015.00023

**Published:** 2015-04-14

**Authors:** Mariangela Morlando, Monica Ballarino, Alessandro Fatica

**Affiliations:** ^1^Department of Biology and Biotechnology, Sapienza University of Rome, Rome, Italy

**Keywords:** lncRNA, leukemia, cancer, hematopoiesis, blood cells

## Abstract

Long non-coding RNAs (lncRNAs) are important regulators of gene expression that influence almost every step in the life cycle of genes, from transcription to mRNA splicing, RNA decay, and translation. Besides their participation to normal physiology, lncRNA expression and function have been already associated to cancer development and progression. Here, we review the functional role and mechanisms of action of lncRNAs in normal hematopoiesis and how their misregulation may be implicated in the development of blood cell cancer, such as leukemia.

## Introduction

**Figure 1:**
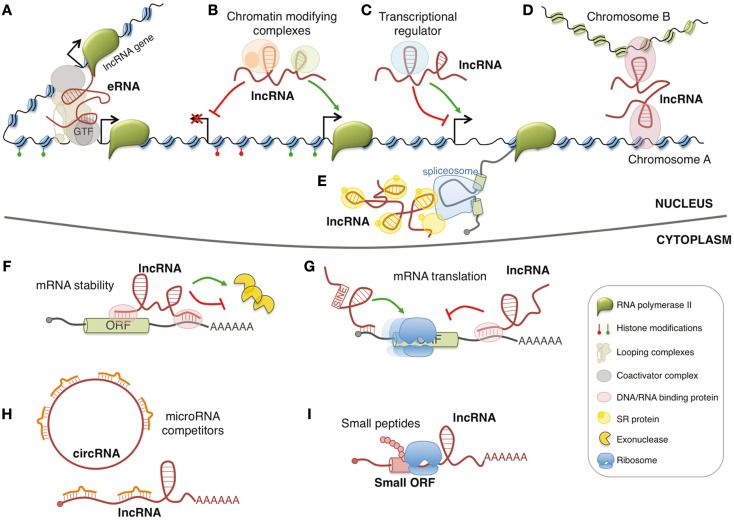
**Models of lncRNA functions**. Nuclear lncRNAs can regulate transcription by acting as enhancer RNA (eRNA) **(A)**, by recruiting chromatin modifying complexes **(B)**, or by regulating transcription factors activity **(C)**. Moreover, they can regulate gene expression by acting on the spatial conformation of chromosomes **(D)** or by influencing pre-mRNA splicing **(E)**. Cytoplasmic lncRNAs can regulate mRNA expression by regulating mRNA stability **(F)**, mRNA translation **(G)**, or by competing for microRNA binding **(H)**. In addition, few lncRNAs contain small open reading frames (ORFs) that can be translated in biological active small peptides **(I)**.

In the last decade, the family of regulatory RNAs has undergone a sudden expansion with the discovery and the functional characterization of long non-coding RNAs (lncRNAs). LncRNAs generally indicate ribonucleic molecules longer than 200 nucleotides without defined open reading frames. To date, the number of human lncRNAs is in the range of ~10,000 transcripts and, for some of them, the involvement in important regulatory circuits of gene expression has been identified (1). However, the majority of lncRNAs remains uncharacterized, and the actual proportion of lncRNAs that might have biological function is hotly debated. LncRNAs are usually classified by their position relative to coding genes and are usually defined as sense, antisense, or intronic, if they overlap with another transcript, bidirectional when their transcription is initiated in close proximity and opposite orientation to a neighboring coding transcript, and intergenic or intervening (known as lincRNAs) if they lie between two genes. In addition, lncRNAs can be also produced from enhancer or promoter regions, known as eRNAs and pRNAs (or PROMPTs), respectively. LncRNAs may act by different mechanisms in both nuclear and cytoplasmic compartments (Figure 1). In particular, they can influence many cellular processes such as spatial conformation of chromosomes, chromatin and DNA modifications, RNA transcription, pre-mRNA splicing, mRNA degradation, and mRNA translation (2). Furthermore, few of them can also produce small biological active peptides (3).

In the last few years, a large amount of sequence data have been generated, which allowed the identification of thousands of lncRNAs expressed in different cell types and stages of development (4). Different studies have already shown that changes in lncRNA expression may contribute to the development and progression of human diseases, including cancer. Here, we review the role of lncRNAs in normal and malignant hematopoiesis, with an emphasis on leukemia, and discuss expectations and challenges in using these RNA molecules as biomarkers and therapeutic targets in leukemia.

## Long Non-Coding RNAs in Normal Hematopoiesis

Several lncRNAs have been already found in the development of blood cells, even if the majority has not yet been functionally characterized. One of the first to be identified was the Eosinophil Granule Ontogeny lncRNA EGO (5). This non-coding transcript was identified in eosinophil differentiation of CD34+ hematopoietic progenitor cells, where it stimulates differentiation and mature cell function by regulating eosinophil granule protein expression at the transcription level (5). However, its mode of action has not been described yet. Subsequently, it was discovered “antisense to PU.1,” a lncRNA, which is antisense to the master hematopoietic transcriptional factor PU.1 and that negatively regulates its expression (6). Generally, PU.1 expression levels are critical for normal hematopoietic development and suppression of leukemia. According to their model, the author suggested that “antisense to PU.1,” lncRNA would keep PU.1 expression levels from being too high by negatively regulating PU.1 mRNA translation. Notably, even though the precise mechanism of action was not characterized, this was one of the first demonstrations of antisense lncRNA acting in the cytoplasm by inhibiting translation. The advent of the first lncRNA catalogs produced by the FANTOM and ENCODE international research consortiums and the development of high-throughput technologies for measuring lncRNAs expression have produced a great boost to the identification of lncRNA from various cell types, including blood cells. HOTAIRM1 was identified by microarray analysis as one of the lncRNAs induced during all-trans retinoid acid (ATRA)-mediated granulocytic differentiation of APL cell lines (7). This lncRNA is one of the numerous lncRNAs produced from the HOXA cluster and its knockdown attenuated the expression of different HOXA genes, which are important transcriptional regulators of normal and malignant hematopoiesis. HOTAIRM1 modulates the levels of several granulocytic differentiation genes (7, 8) and a delay in ATRA-induced granulocytic differentiation was reported upon HOTAIRM1 knockdown (8). Also in this case, the molecular mechanism of HATAIRM1 action is still not known. In 2011, Hu and colleagues report the identification by RNA-sequencing of the “Erythroid ProSurvival lincRNA” (lincRNA-EPS) between about 400 lncRNAs whose expression is modulated during mouse erythroid differentiation (9). LincRNA-EPS promotes terminal differentiation and inhibits apoptosis of mature erythrocytes by inhibiting the pro-apoptotic gene Picard expression through a still not defined mechanism (9). A second high-throughput study by RNA-seq in murine erythropoiesis identified 132 novel lncRNAs with erythroid-restricted expression, many of which are targeted by key erythroid transcription factors (10). Knockdown of 12 of these lncRNAs severely impaired erythroid maturation, suggesting important regulatory functions. One of them is produced from an enhancer region of the BAND3 gene, the major anion transporter of the red cell membrane, and is required for its transcriptional activation acting, very likely, as an enhancer RNA (eRNA) (10). A similar study was performed on mouse erythroblasts, megakaryocytes, megakaryocyte-erythroid precursors, and human erythroblasts (11). In particular, the authors identified several lncRNAs specific for each cell type, many of them regulated by key hematopoietic transcription factors including GATA1 and TAL1. By knocking down 21 of the most abundant murine lncRNAs, they showed that, despite the lack of conservation, 7 of them were required for erythroid terminal differentiation. Furthermore, four of the functional lncRNAs were also found by the independent study of Alvarez-Dominguez et al. (see above). Notably, the majority of the identified lncRNAs were not conserved in human erythroblasts. The general low conservation of lncRNAs may be due to the fact that their tertiary structure is more important than the primary sequence if compared with protein-coding RNAs. Indeed, many lncRNAs function as platform for the assembly of macromolecular complexes (1, 12).

Profiling of lncRNA expression during differentiation of monocytes into dendritic cells (DCs) identified lnc-DC (13). Lnc-DC is a cytoplasmic lncRNA that acts by activating the transcription factor STAT3, which is required for DCs differentiation. In particular, lnc-DC directly binds STAT3 and maintains its active phosphorylated form by blocking its dephosphorylation by Src homology region 2 domain-containing phosphatase-1 (SHP-1).

Emerging data have identified an important contribution of lncRNAs to the development and function of adaptive immune cells. Microarray analysis on purified human and mouse CD8^+^ T-cells identified 29 lncRNAs specific for these lymphocytes, and, more importantly, 21 out of them were modulated during memory T-cell differentiation, 81 during lymphocyte activation, and 4 during both transitions. A more comprehensive analysis of lncRNA expression during T-cell development was performed from 42 different T-cell types at various developmental and differentiation stages by using RNA-seq (14). This study identified 1,524 genomic regions expressing lncRNAs that are much more specific for lineage or developmental stage compared to mRNAs. T-cell-specific transcription factors, such as T-bet, STAT4, GATA-3, and STAT6, were shown to be largely involved in the specific expression of the identified lincRNAs in different T-cells lineages. Furthermore, many lncRNAs are adjacent to genes encoding proteins critical to T-cell function. One of them, LincR-Ccr2-5’AS, controls the expression of several chemokine receptors and, eventually, the migration of T-cells. However, also in this case the mechanism of action was not identified. More recently, a second study performed a lncRNA profiling in human T- and B-lymphocytes at different differentiation stages by RNA-seq and identified over 500 previously unknown lncRNAs (15). One of them, linc-MAF4, regulates the expression of MAF, a transcription factor required for T-cell function, through the recruitment of chromatin modifiers.

LncRNAs are also involved in the regulation of the innate immune and inflammatory responses, as reviewed in detail elsewhere (16).

## Long Non-Coding RNAs in Leukemia

Defects in cell differentiation and uncontrolled proliferation are a hallmark of leukemia. Thus, every lncRNA that is involved in gene expression control during the development of blood cells, if deregulated, might contribute to the development and the progression of cancer. Since their discovery, lncRNAs have been profiled by various tumor types, including leukemia and other hematopoietic malignancies. Moreover, lncRNA expression has been already correlated with diagnostic and prognostic factors. However, only few lncRNAs have been directly involved in the tumorigenic processes of blood cell cancers.

Human T cell acute lymphoblastic leukemia (T-ALL) is generally characterized by mutations that activate the NOTCH1 signaling (17). The lncRNA LUNAR1 (leukemia-induced non-coding activator RNA) is a NOTCH-regulated oncogenic lncRNA in T-ALL (18) that promotes T-ALL cell growth due to its ability to enhance IGF1R mRNA expression and sustains IGF1 signaling. In particular, LUNAR1 acts as eRNA by recruiting the mediator complex on the IGF1R promoter to activate, eventually, its transcription. Notably, T-ALL cells with lower LUNAR1 levels showed a significant loss of proliferation potential in xenograft assay (18).

A microarray analysis of lncRNAs from chronic myeloid leukemia (CML) with Bcr–Abl translocation identified the Beta Globin Locus 3 (BGL3) lncRNA (19). This lncRNA positively regulates the level of the tumor suppressor PTEN by acting as a competing endogenous RNA (ceRNA) (20). Notably, lncRNA-BGL3 expression decreased the survival of Bcr–Abl-positive K562 leukemic cells and stimulated the apoptosis induced by imatinib, a tyrosine kinase inhibitor used in the treatment of Bcr–Abl CML. Moreover, its expression affected Bcr–Abl-induced tumorigenesis in xenograft mouse model (19). Another relevant lncRNA in Bcr–Abl CML is the imprinted H19 lncRNA, which plays important roles in embryonal development and growth control (21). This lncRNA was found upregulated in many different cancers, including Bcr–Abl CML, and predicted to be an oncogenic lncRNA. Moreover, in Bcl–Abl CML cells, H19 is positively regulated by the oncogene c-Myc (21). Down-regulation of H19 levels affected cell proliferation and attenuated tumor formation in xenograft assay (21). Notably, in muscle cells and different solid tumors, H19 has been shown to inhibit the oncosuppressor let-7 microRNA family, thus acting as a ceRNA (22–24). Since microRNAs play an important role in blood cell development and leukemia, it is easy to predict that, in the future, other lncRNAs regulating microRNA function in the blood system will be identified.

Another lncRNA involved in acute myeloid leukemia (AML) is IRAIN (25), which is produced from the insulin-like growth factor type I receptor (IGF1R) imprinted locus. Aberrant expression of IGF1R promotes AML cell growth through the PI3K/Akt signaling pathway. IRAIN is transcribed antisense to IGF1R and is involved in the formation of a long-range intrachromosomal interaction between the IGF1R promoter and a distant intragenic enhancer (25). Moreover, IRAIN is down-regulated in leukemia cell lines and in patients with high-risk AML.

The well-known X-inactive specific transcript Xist lncRNA, an important regulator of X-chromosome inactivation in mammals, is aberrantly expressed in different human cancer, including leukemia (26). Xist provided one of the first examples of an lncRNA that is directly involved in the formation of repressive chromatin and in addition to dosage compensation it may also have an important role in cell differentiation. In mouse hematopoietic stem cells, both the homozygous and heterozygous conditional deletion of Xist resulted in the development of blood cell cancers and in accelerated death, further suggesting that alteration in the X inactivation process contributes to tumorigenesis (27).

Long non-coding RNAs have been profiled from patients with citogenetically normal (CN) AML (28). Distinctive lncRNA profiles were found to be associated with specific mutations, such as mutations in the NPM1, CEBPA, IDH2, ASXL1, and RUNX1 genes, and internal tandem duplications in the FLT3 gene (FLT3-ITD). More importantly, lncRNA expression correlated with treatment response and survival (28). One of the lncRNA that is specifically upregulated in (CN) AML with CEBPA mutation is the oncogenic urothelial carcinoma-associated 1 lncRNA (UCA1). UCA1 was found upregulated in different solid tumors and its down-regulation affects proliferation of AML cells (29). An lncRNA profiling was also performed in B-acute lymphoblastic leukemia (B-ALL) (30). Also in B-ALL, lncRNA expression correlated with cytogenetic abnormalities and survivals of B-ALL patients. Moreover, the lncRNA B-ALL-associated long RNAs-2 (BARL-2) was identified as a modulator of the response to corticosteroid treatment, which is used in B-ALL therapy. Therein, these studies suggest that, similarly to microRNAs, lncRNAs might be utilized as diagnostic and prognostic markers in leukemia.

## Conclusion

In the next future, we will witness a comprehensive identification and functional analyzes of lncRNAs expressed during all stages of hematopoiesis and in multiple species, which will contribute to better defining the role of these molecules in blood cell development. Thus, it seems safe to predict that many new functional lncRNAs will be identified. Furthermore, lncRNAs might have diagnostic applications, with changes in their expression already associated with different classes of leukemia, regardless of function.

It is very likely that the established molecular circuitries known to control blood cell differentiation will need to be reevaluated in the light of the contribution of this complex class of non-coding transcripts. However, the identification of functional molecules and, above all, the mechanisms by which they can regulate gene expression is still challenging for the field. Many lncRNAs, especially those acting *in cis*, are present in only few copies per cell and might not be considered in functional studies. Moreover, different functions might be active in different cells and at different developmental stages. LncRNAs can be very long, even several thousand nucleotides, which fold in complex three-dimensional structures to act as molecular scaffolds. Indeed, a single lncRNA molecule might carry out a large number of functions within the cells by interacting and cooperate with different cell-specific interactors (both proteins and nucleic acids) or by localizing in specific compartments. A large amount of sequence data have been generated in last few years, which already allowed the identification of thousands of lncRNAs expressed in different stages of hematopoietic development but most studies merely identify functional lncRNAs without explaining how these molecules act within the cell. This has further generated the need to develop new experimental tools to identify and analyze lncRNA mechanisms of action. Furthermore, targeted deletion studies in primary cells, or animal models when applicable, will be necessary to assess the specific functions of lncRNAs involved in normal and malignant hematopoiesis.

Another important aspect to consider is the fact that, despite the advantages of RNA-seq in terms of sensitivity and accessibility, the assembly of transcript models from short reads is still limited and the bioinformatic tools utilized often provide different outcomes from same raw data. Therein, the lists of lncRNAs identified in different studies from the same cell type do not always coincide. In the next future, the development of technologies for long reads sequencing that minimize the bioinformatic assembly of novel transcripts will be of great help to standardize and unify the many different existing lists of lncRNAs.

In conclusion, similarly to the most famous microRNAs, lncRNAs will become subject of several studies in normal and malignant hematopoiesis that, hopefully, will help to determine the therapeutic significance of lncRNAs in leukemia.

## Conflict of Interest Statement

The authors declare that the research was conducted in the absence of any commercial or financial relationships that could be construed as a potential conflict of interest.
